# Supplementary figures and images for: Slow-wave sleep drives sleep-dependent renormalization of synaptic AMPA receptor levels in the hypothalamus
Source: PLoS Biol. 2024 Aug 20;22(8):e3002768. doi: 10.1371/journal.pbio.3002768 (PMC11364421; doi:10.1371/journal.pbio.3002768)

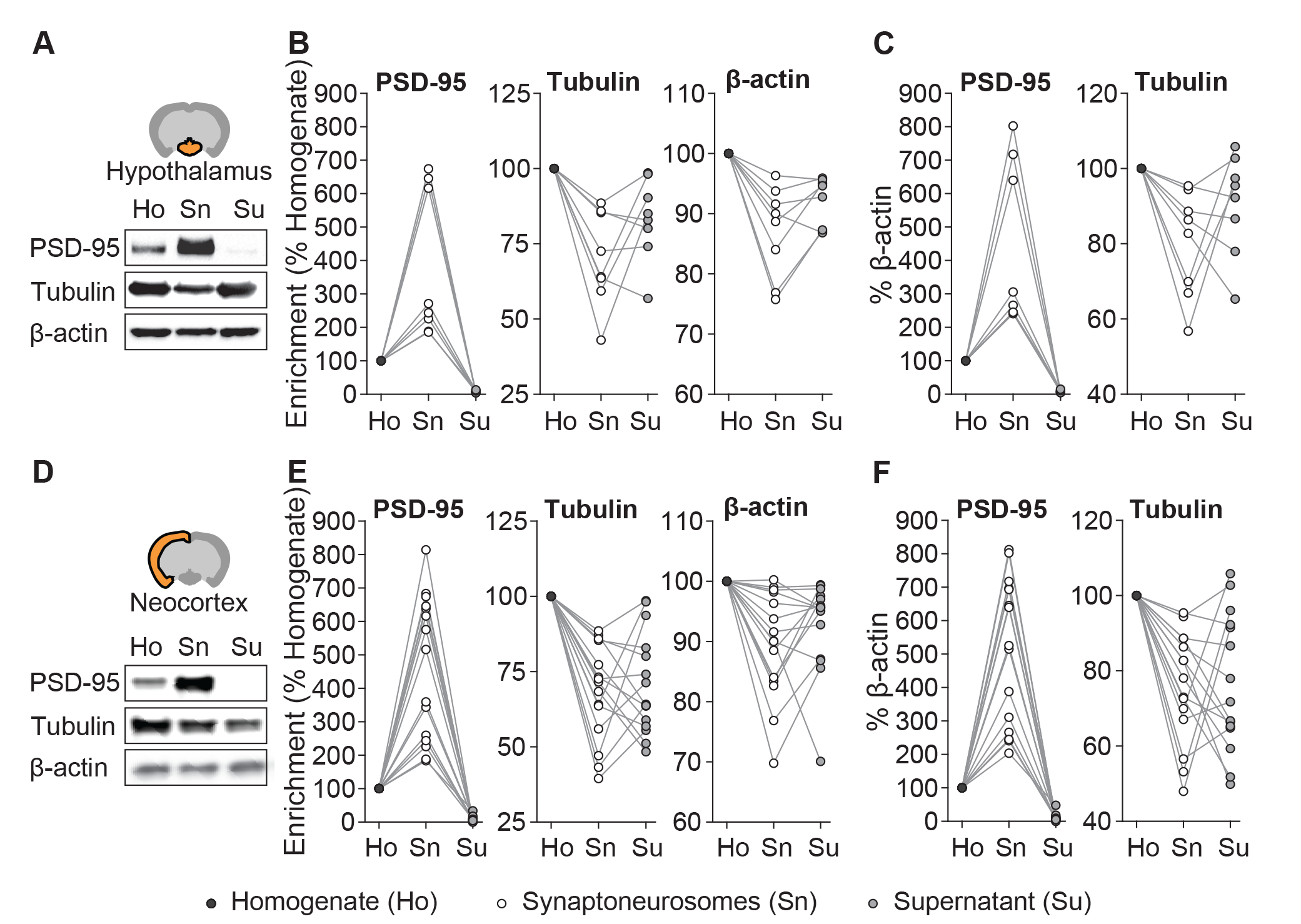

Supplement: S1 Fig — Representative western blots showing homogenate (Ho), synaptoneurosomes (Sn), and supernatant (Su) fractions from (A) hypothalamus and (D) neocortex. (B, E) Quantification of PSD-95, tubulin, and ß-actin. Integrated density values were normalized to values of homogenates (set to 100%) for samples from each animal. (C, F) Quantification of PSD-95 and tubulin relative to ß-actin density were normalized to values of homogenates (set to 100%). Circles represent samples from individual animals (hypothalamus, n = 8 rats; neocortex, n = 15 rats); the underlying data sets are available in an online supporting file (S1 Data). (TIF) [file pbio.3002768.s001.tif]

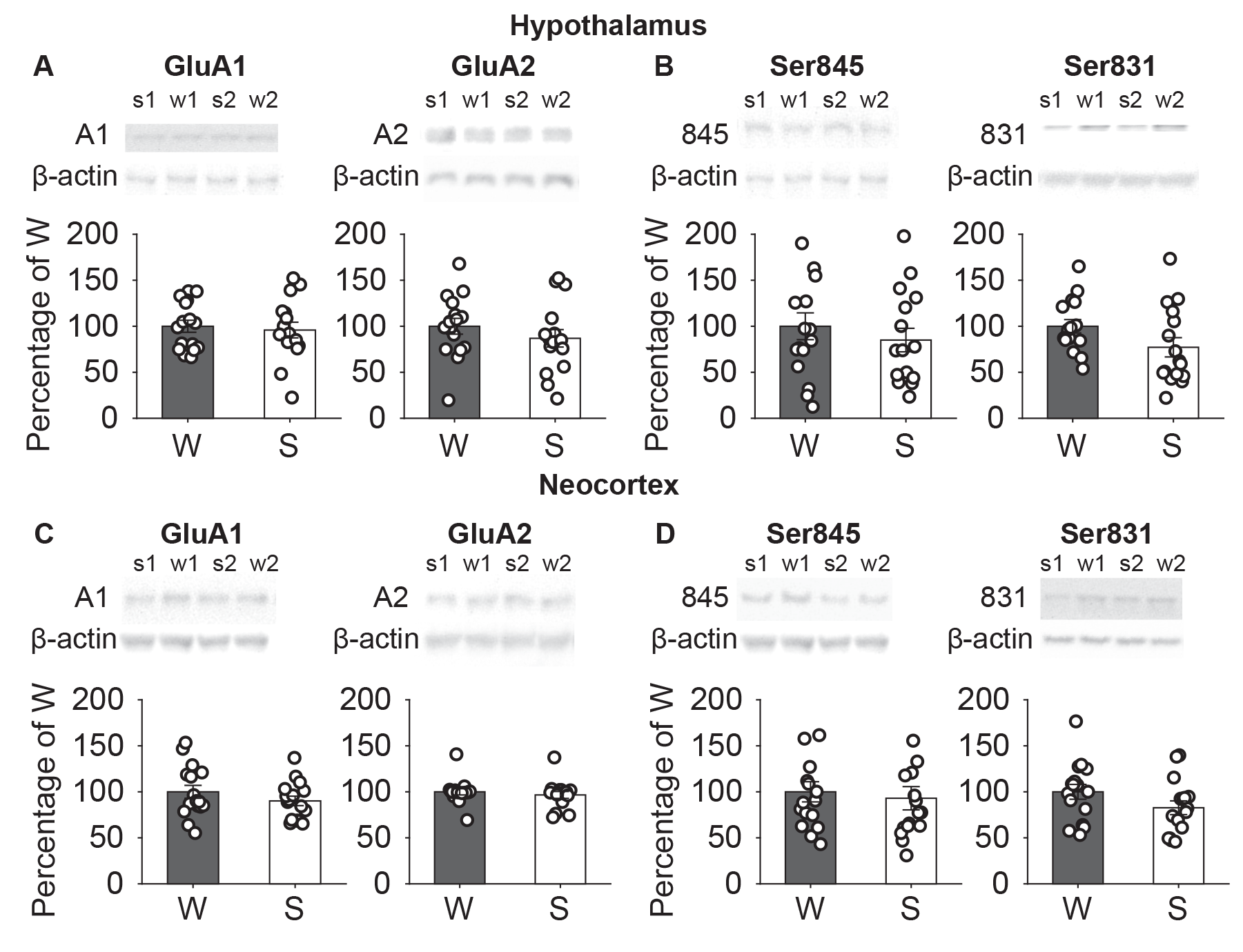

Supplement: S2 Fig — (A) Levels of GluA1- (left) and GluA2-containing AMPARs (right) and (B) of GluA1 phosphorylated at Ser845 (left) and at Ser831 (right) in hypothalamus and (C, D) neocortex. Mean ± SEM normalized AMPAR levels are shown with the mean for the Wake group set to 100%. On top, 2 example immunoblots are shown for each group (s1, s2, w1, w2; GluA1, GluA2, phospho-Ser845, and phospho-Ser831 bands were normalized with reference to the corresponding β-actin band in the same sample, the latter serving as loading control). There were no significant differences between groups for any measure; the underlying data sets are available in an online supporting file (S1 Data). (TIF) [file pbio.3002768.s002.tif]

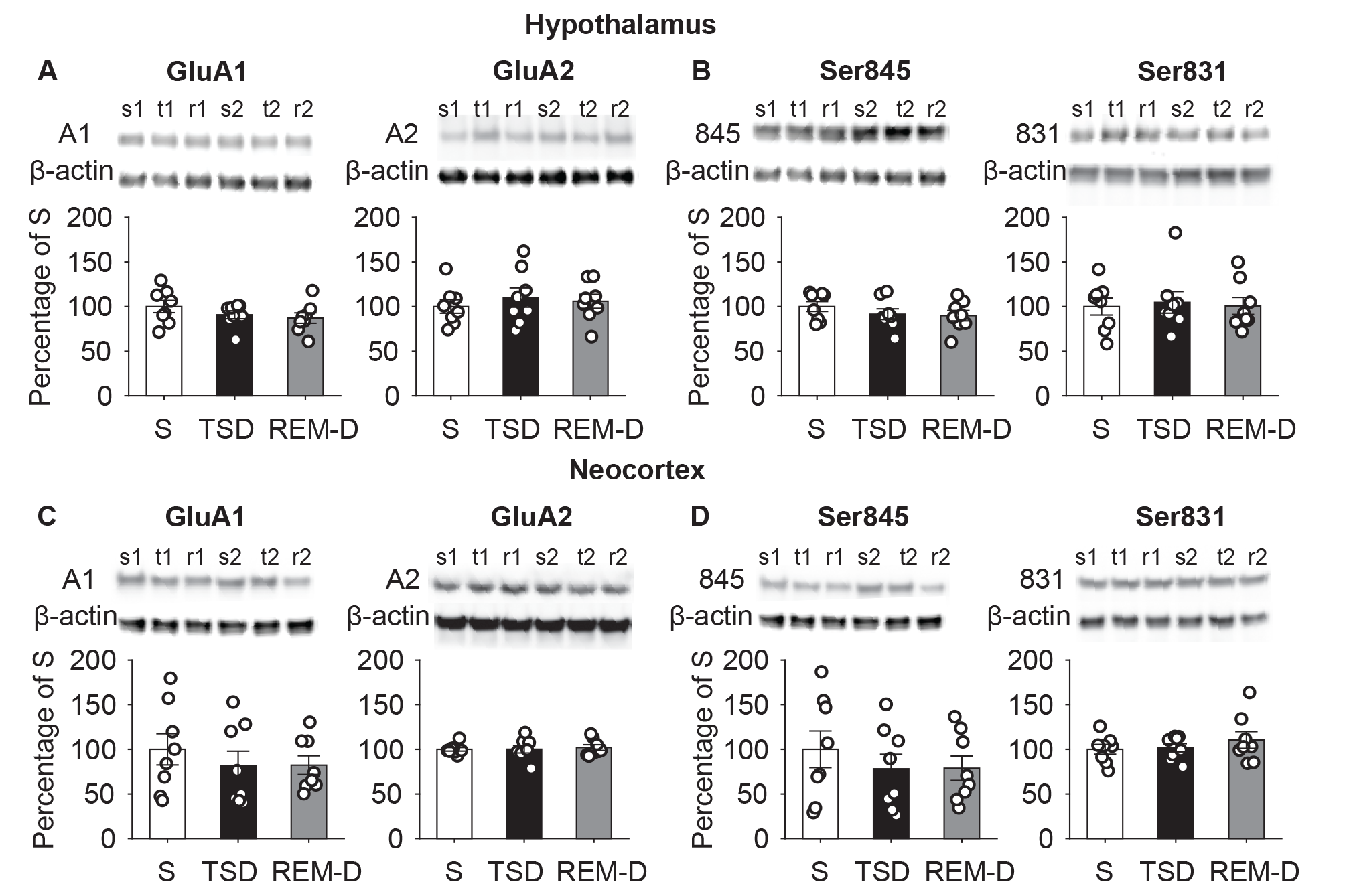

Supplement: S3 Fig — (A) Levels of GluA1- (left) and GluA2-containing AMPARs (right) and (B) of GluA1 phosphorylated at Ser845 (left) and at Ser831 (right) in hypothalamus and (C, D) neocortex. Mean ± SEM normalized AMPAR levels are shown with the mean for the Sleep control group set to 100%. On top, 2 example immunoblots are shown for each group (s1, s2, t1, t2, r1, r2; GluA1, GluA2, phospho-Ser845, and phospho-Ser831 bands were normalized with reference to the corresponding β-actin band in the same sample, the latter serving as loading control). There were no significant differences between groups for any measure; the underlying data sets are available in an online supporting file (S1 Data). (TIF) [file pbio.3002768.s003.tif]

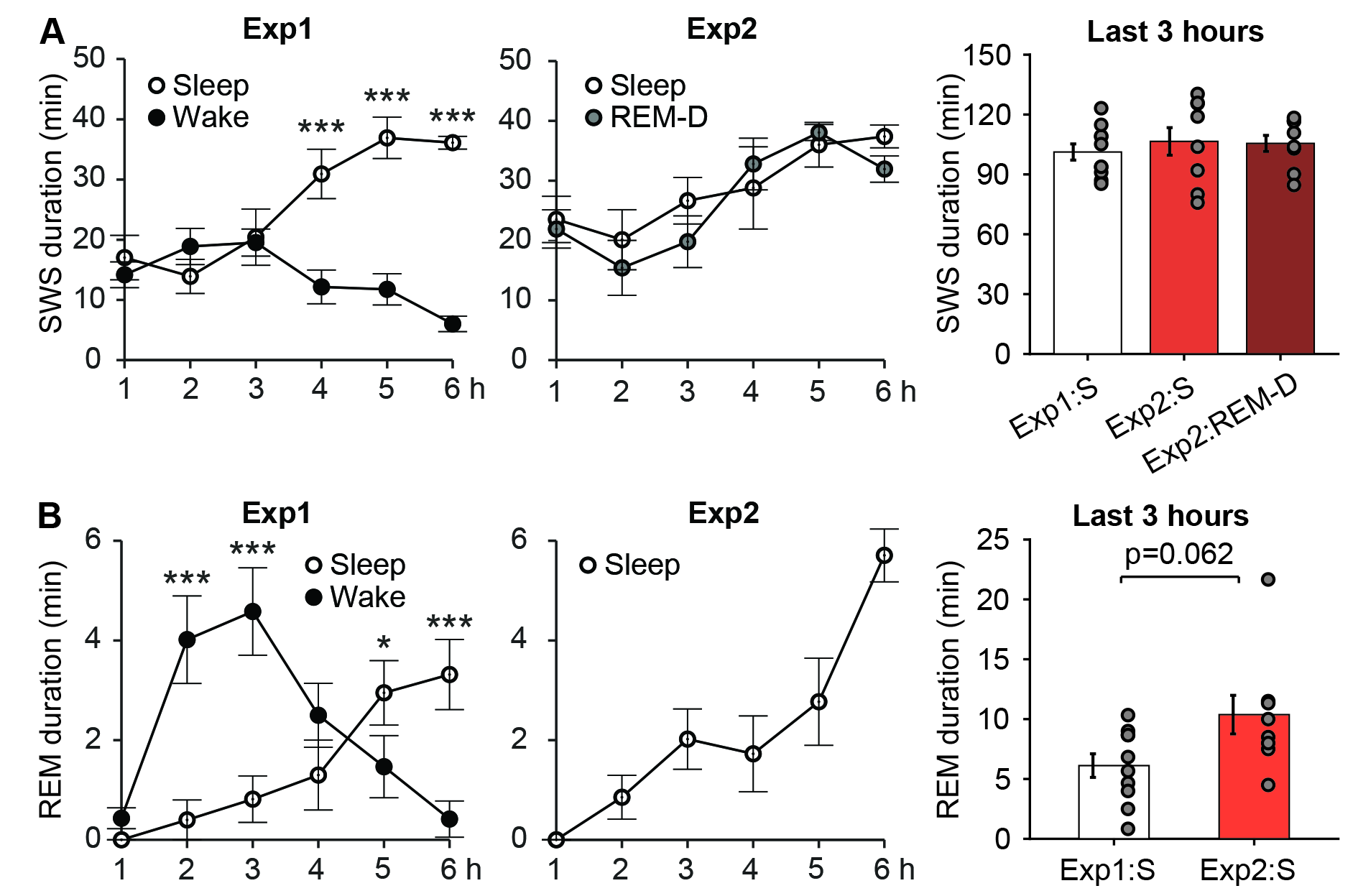

Supplement: S4 Fig — Amount of (A) SWS and (B) REM sleep in minutes during each hour of the 6-hour recording session in the Sleep and Wake groups of experiment 1 (left panels) and in the Sleep and, as applicable, REM sleep-deprivation (REM-D) groups of experiment 2 (midline panels); SWS and REM sleep duration in minutes during the final 3 hours in the Sleep (Exp1:S and Exp2:S, respectively) and, as applicable, REM-D groups in experiments 1 and 2 (right panels); *** p < 0.001, unpaired t tests. Note that overall, the sleep ratios are very much comparable between the respective groups of experiments 1 and 2; the underlying data sets are available in an online supporting file (S1 Data). (TIF) [file pbio.3002768.s004.tif]
