# Supplementary material for: Slow-wave sleep drives sleep-dependent renormalization of synaptic AMPA receptor levels in the hypothalamus
Source: PLoS Biol. 2024 Aug 20;22(8):e3002768. doi: 10.1371/journal.pbio.3002768 (PMC11364421; doi:10.1371/journal.pbio.3002768)
Supplement: S1 Table — (DOCX) [file pbio.3002768.s005.docx]

**S1 Table**. Correlations between sleep parameters of interest and levels of GluA1-containing AMPARs in hypothalamus and neocortex in experiment 1.

|  | **Hypothalamic GluA1** | | **Cortical GluA1** | |
| --- | --- | --- | --- | --- |
|  | **1st 3 hours** | **2nd 3 hours** | **1st 3 hours** | **2nd 3 hours** |
| **SWS time** | r = 0.0432 | r = -0.7290 * | r = 0.3977 | r = 0.4124 |
| **Spindle density** | r = 0.5090 | r = 0.6791 * | r = 0.1343 | r = 0.0113 |
| **SWA power** | r = -0.3202 | r = -0.4853 | r = -0.1562 | r = -0.1512 |
| **SWA energy** | r = -0.3999 | r = -0.5120 | r = 0.4443 | r = 0.1336 |
| **SO density** | r = 0.2776 | r = -0.0603 | r = -0.0488 | r = -0.1550 |
|  |  |  |  |  |
| **REM time** | r = 0.1309 | r = -0.6471 * | r = 0.2526 | r = 0.5593 |
| **REM theta power** | r = 0.2554 | r = -0.5701 | r = 0.1446 | r = 0.5033 |
| **REM theta energy** | r = 0.1314 | r = -0.8740 *** | r = 0.2545 | r = 0.4383 |

Pearson’s correlation coefficients were calculated separately for the first and second half of the 6-hour period prior to AMPAR assessment in the animals of the Sleep group, n = 10. * p ≤ 0.05; *** p < 0.001, uncorrected for multiple comparisons. Sleep parameters of interest for slow-wave sleep (SWS) comprised time spent in SWS, spindle density, slow-wave activity (SWA), including the average power in the 0.1-4 Hz frequency band and the energy (i.e., power over time), and slow oscillation (SO) density. Parameters of interest for REM sleep comprised time spent in REM sleep and the average power and energy in the 4-8 Hz theta frequency band.
