# Supplementary material for: Slow-wave sleep drives sleep-dependent renormalization of synaptic AMPA receptor levels in the hypothalamus
Source: PLoS Biol. 2024 Aug 20;22(8):e3002768. doi: 10.1371/journal.pbio.3002768 (PMC11364421; doi:10.1371/journal.pbio.3002768)

Fig.1C

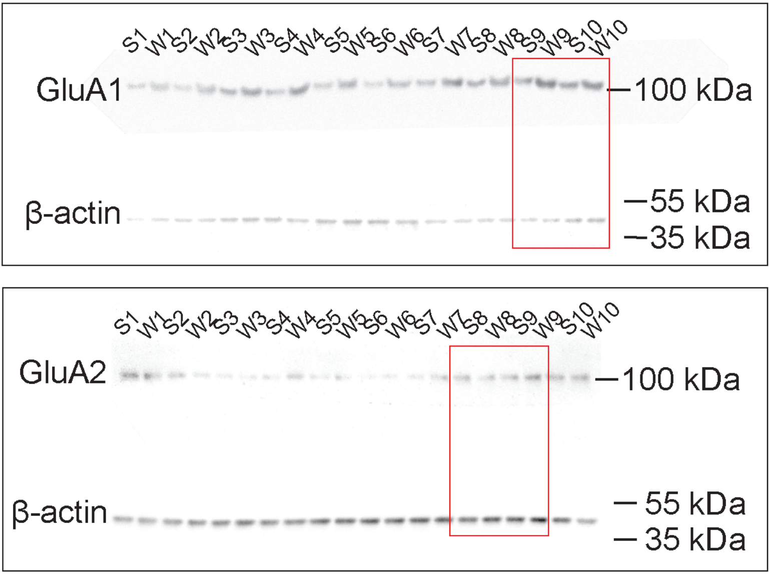
S1 W1 S2 W2 S3 W3 S4 W4 S5 W5 S6 W6 S7 W7 S8 W8 S9 W9 S10 W10
GluA1
100 kDa
β-actin
55 kDa
35 kDa
S1 W1 S2 W2 S3 W3 S4 W4 S5 W5 S6 W6 S7 W7 S8 W8 S9 W9 S10 W10
GluA2
100 kDa
β-actin
55 kDa
35 kDa

Fig.1D

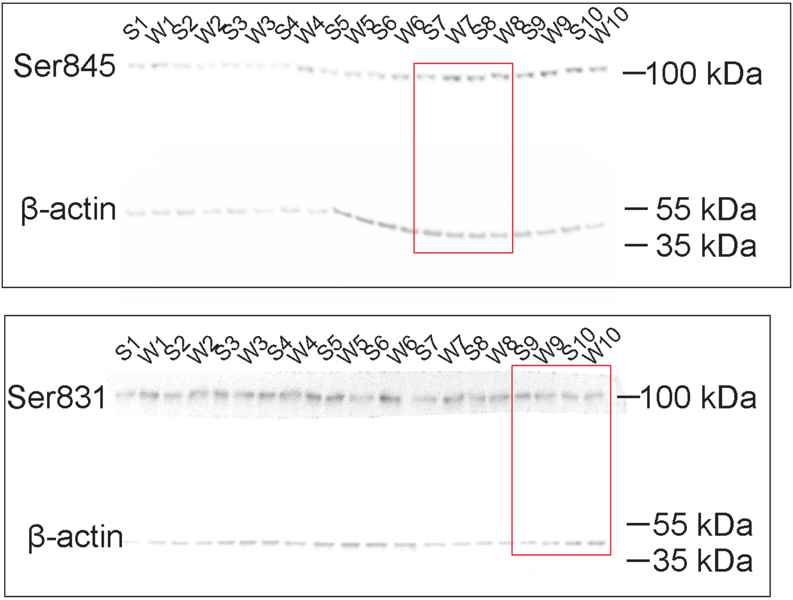
S1 W1 S2 W2 S3 W3 S4 W4 S5 W5 S6 W6 S7 W7 S8 W8 S9 W9 S10 W10
Ser845
100 kDa
β-actin
55 kDa
35 kDa
S1 W1 S2 W2 S3 W3 S4 W4 S5 W5 S6 W6 S7 W7 S8 W8 S9 W9 S10 W10
Ser831
100 kDa
β-actin
55 kDa
35 kDa

Fig.1E

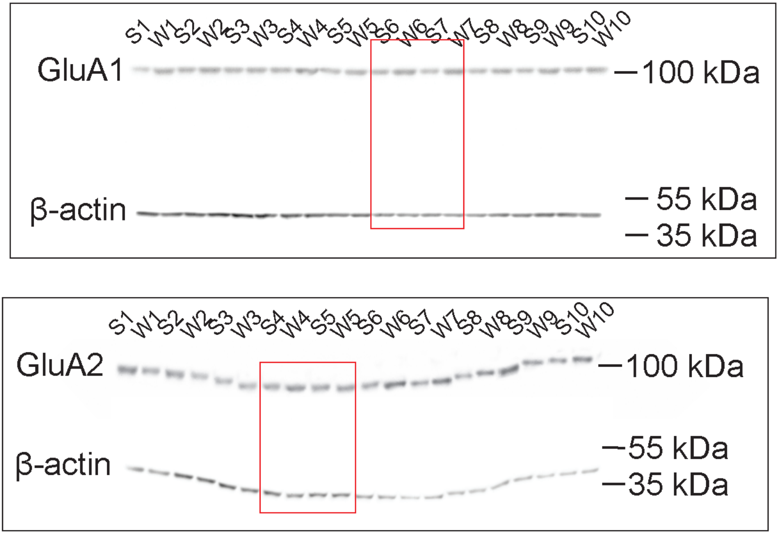
S1 W1 S2 W2 S3 W3 S4 W4 S5 W5 S6 W6 S7 W7 S8 W8 S9 W9 S10 W10
GluA1
100 kDa
β-actin
55 kDa
35 kDa
S1 W1 S2 W2 S3 W3 S4 W4 S5 W5 S6 W6 S7 W7 S8 W8 S9 W9 S10 W10
GluA2
100 kDa
β-actin
55 kDa
35 kDa

Fig.1F

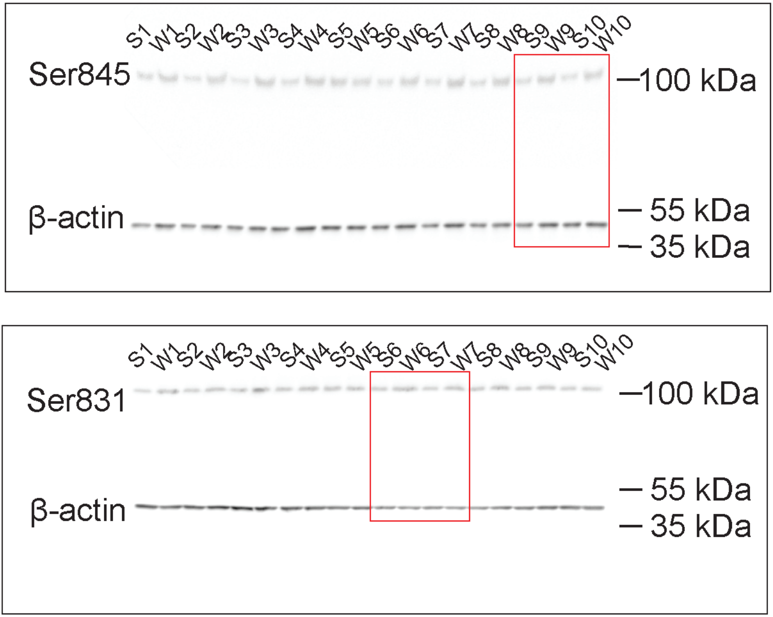
S1 W1 S2 W2 S3 W3 S4 W4 S5 W5 S6 W6 S7 W7 S8 W8 S9 W9 S10 W10
Ser845
100 kDa
β-actin
55 kDa
35 kDa
S1 W1 S2 W2 S3 W3 S4 W4 S5 W5 S6 W6 S7 W7 S8 W8 S9 W9 S10 W10
Ser831
100 kDa
β-actin
55 kDa
35 kDa

Fig.2C

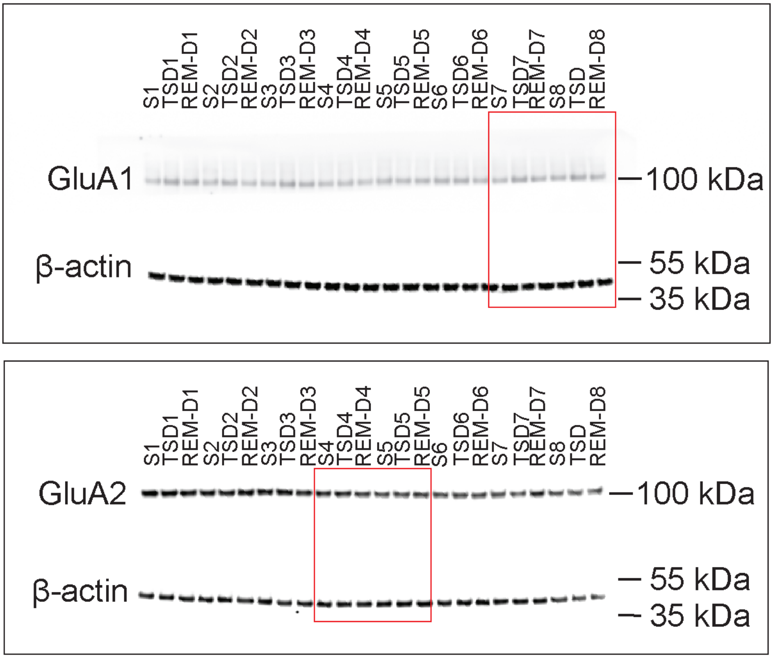
S1
TSD1
REM-D1
S2
TSD2
REM-D2
S3
TSD3
REM-D3
S4
TSD4
REM-D4
S5
TSD5
REM-D5
S6
TSD6
REM-D6
S7
TSD7
REM-D7
S8
TSD
REM-D8
GluA1
β-actin
100 kDa
55 kDa
35 kDa
GluA2
β-actin
100 kDa
55 kDa
35 kDa

Fig.2D

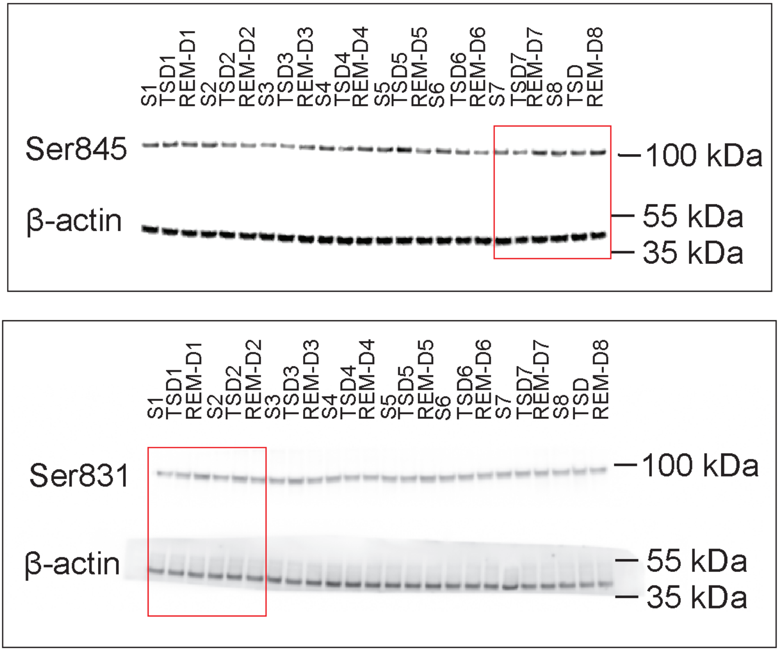
S1
TSD1
REM-D1
S2
TSD2
REM-D2
S3
TSD3
REM-D3
S4
TSD4
REM-D4
S5
TSD5
REM-D5
S6
TSD6
REM-D6
S7
TSD7
REM-D7
S8
TSD
REM-D8
Ser845
β-actin
100 kDa
55 kDa
35 kDa
Ser831
β-actin
100 kDa
55 kDa
35 kDa

Fig.2E

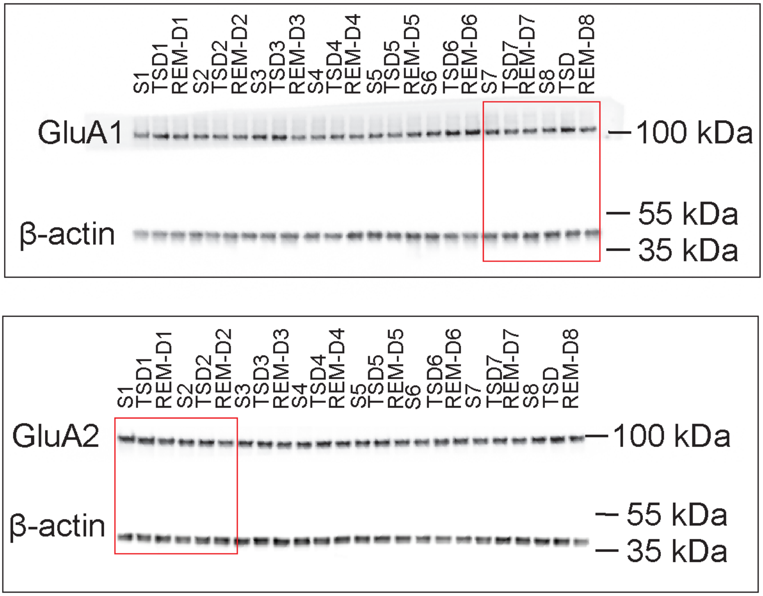
S1
TSD1
REM-D1
S2
TSD2
REM-D2
S3
TSD3
REM-D3
S4
TSD4
REM-D4
S5
TSD5
REM-D5
S6
TSD6
REM-D6
S7
TSD7
REM-D7
S8
TSD
REM-D8
GluA1
β-actin
100 kDa
55 kDa
35 kDa
GluA2
β-actin
100 kDa
55 kDa
35 kDa

Fig.2F

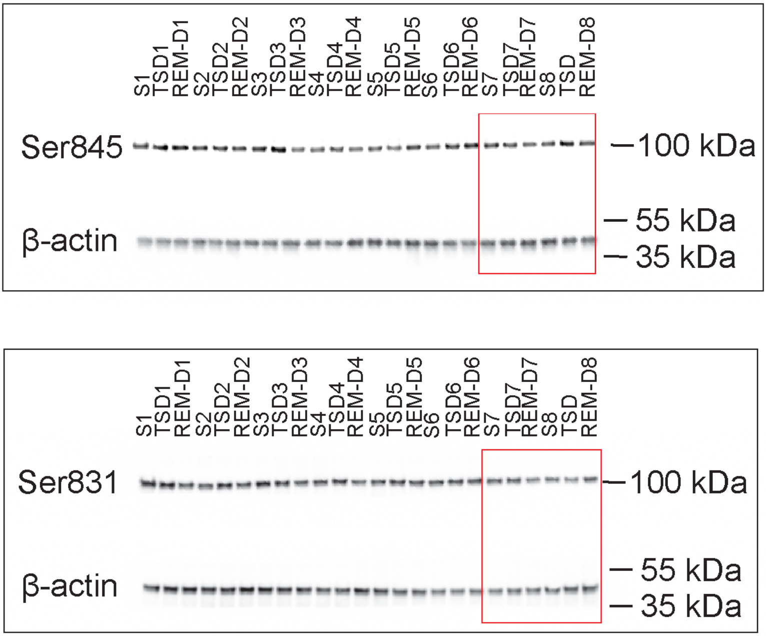
S1
TSD1
REM-D1
S2
TSD2
REM-D2
S3
TSD3
REM-D3
S4
TSD4
REM-D4
S5
TSD5
REM-D5
S6
TSD6
REM-D6
S7
TSD7
REM-D7
S8
TSD
REM-D8
Ser845
β-actin
100 kDa
55 kDa
35 kDa
Ser831
β-actin
100 kDa
55 kDa
35 kDa

Fig.S1A

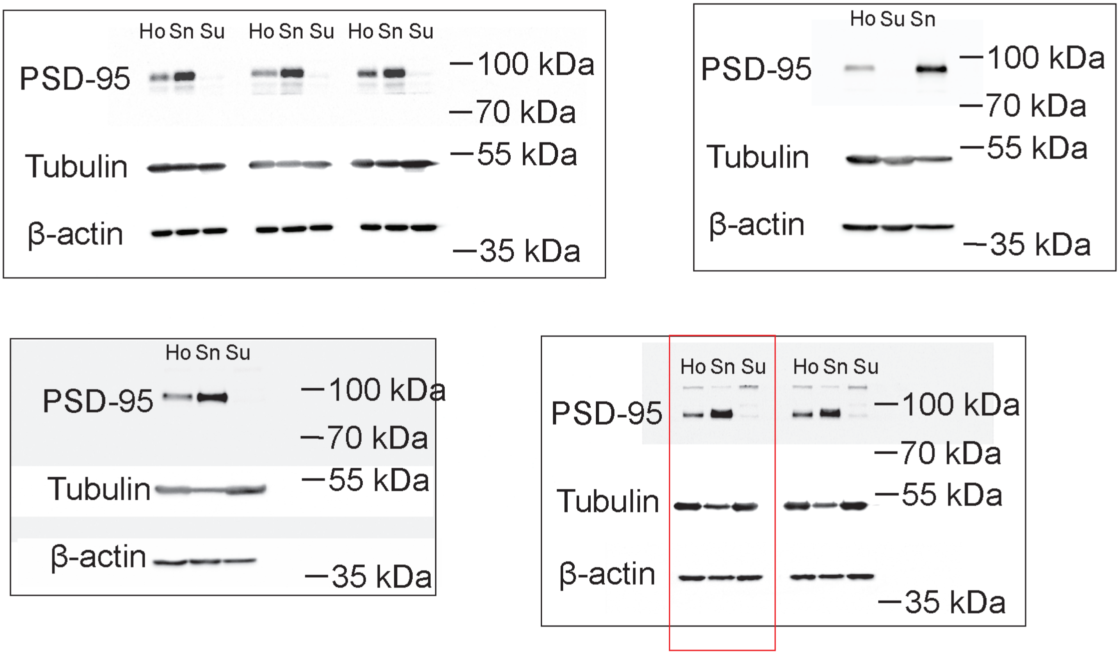

Fig.S1D

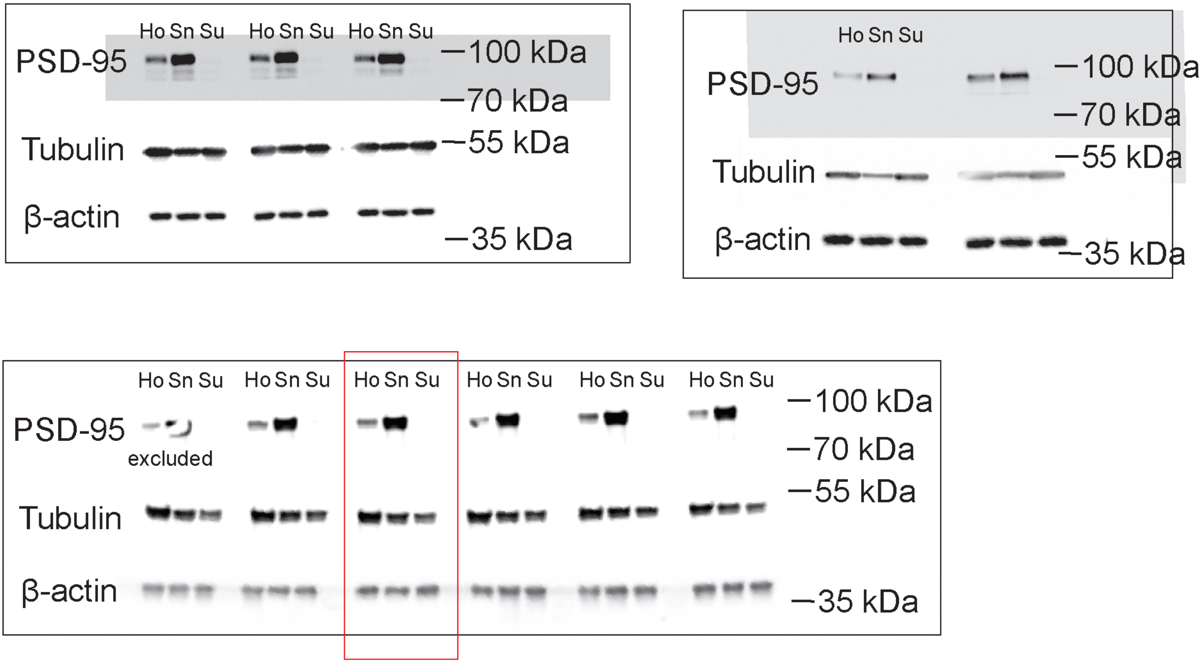

Fig.S2A

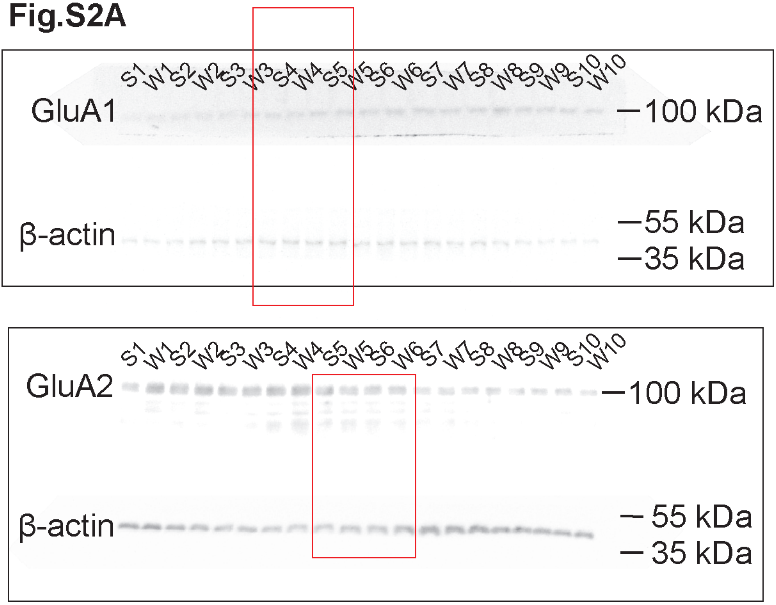

Fig.S2B

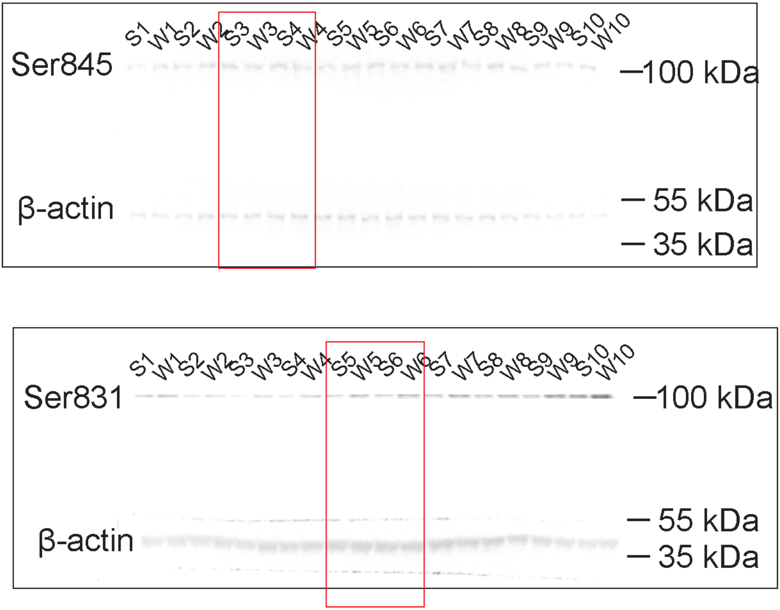

Fig.S2C

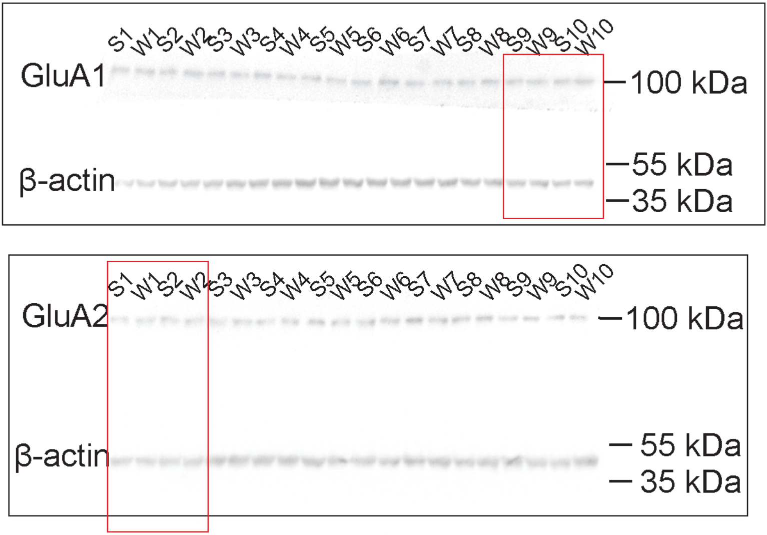

Fig.S2D

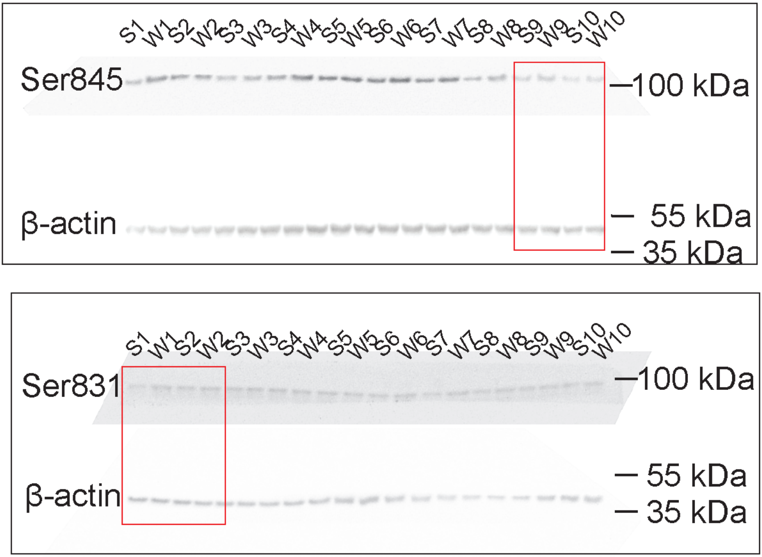

Fig.S3A

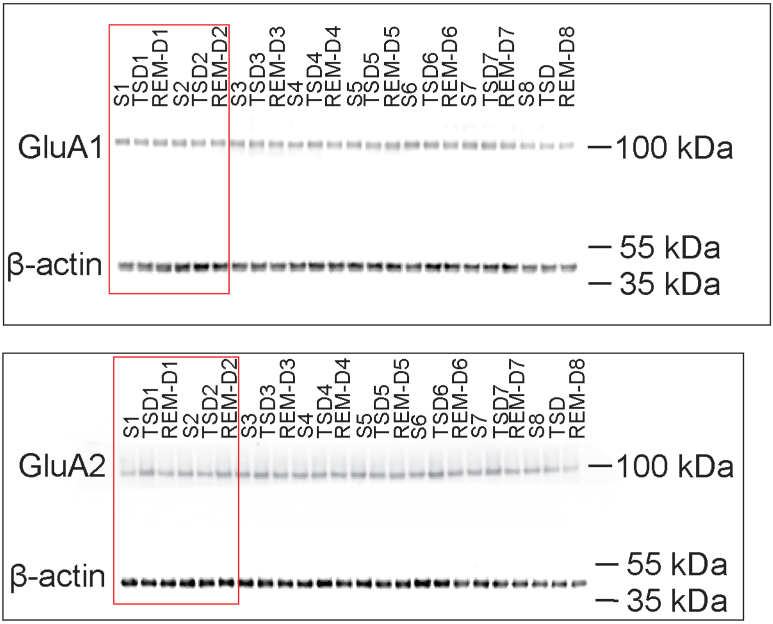

Fig.S3B

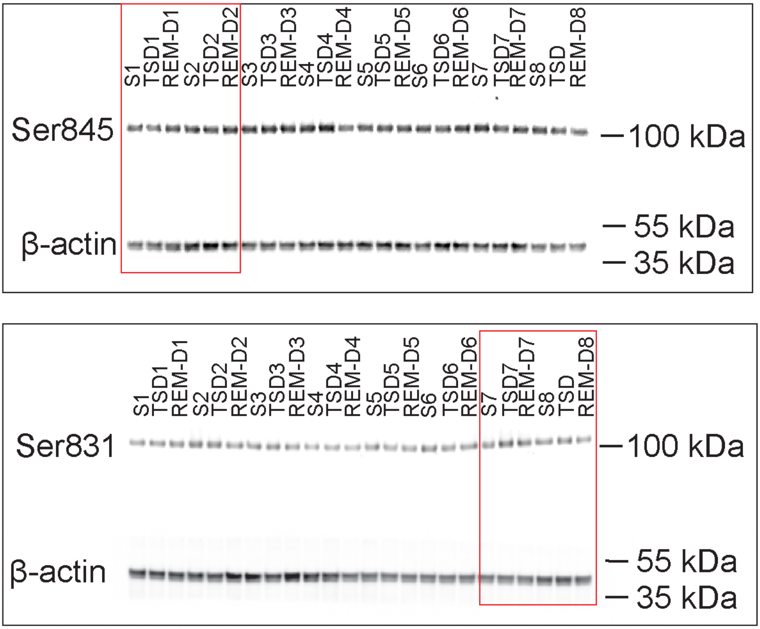

Fig.S3C

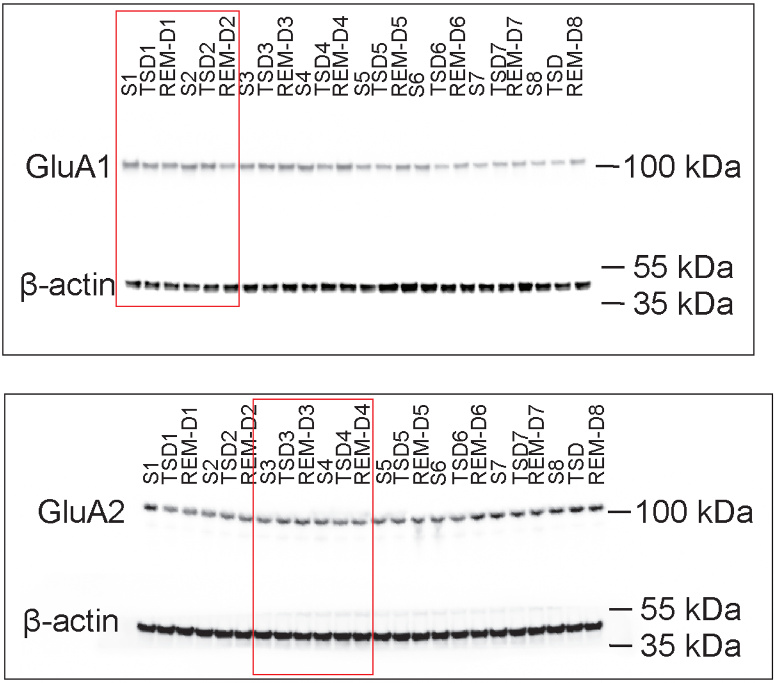

Fig.S3D

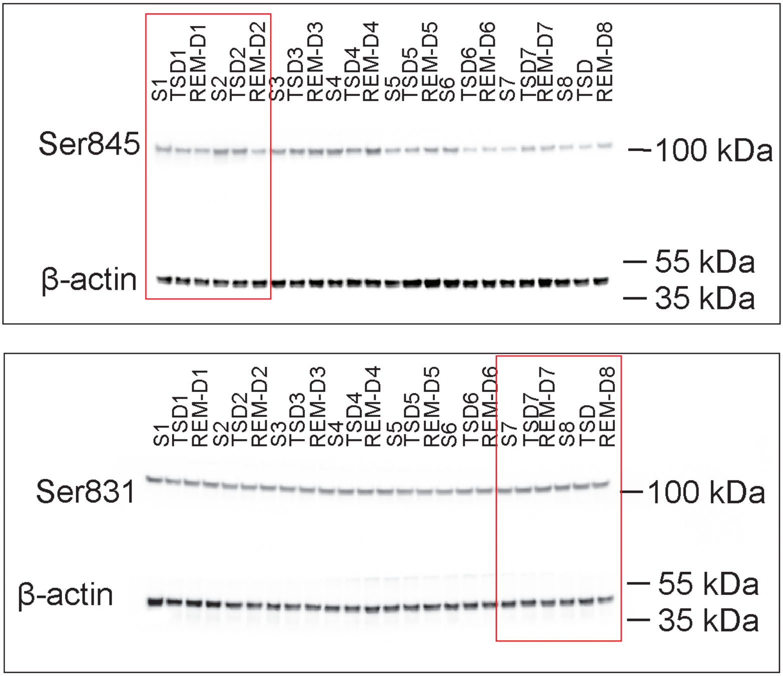

Supplement: S1 Raw Images — (PDF) [file pbio.3002768.s007.pdf]
