# Supplementary material for: Slow-wave sleep drives sleep-dependent renormalization of synaptic AMPA receptor levels in the hypothalamus
Source: PLoS Biol. 2024 Aug 20;22(8):e3002768. doi: 10.1371/journal.pbio.3002768 (PMC11364421; doi:10.1371/journal.pbio.3002768)
Supplement: Attachment — Submitted filename: PBIOLOGY-D-24-00611R3_response to reviewers.pdf [file pbio.3002768.s008.pdf]

Reviewer #1

The work explores whether the synaptic strength reductions known to happen sleep may occur in not just regions affected by sleep, but also the hypothalamus which controls sleep. They assess protein levels and phosphorylation of synaptosomes and do indeed find that especially the GluA1 subunit and it's phosphorylation are reduced, suggesting possibly reduced synaptic transmission in that structure.

1. The data in the first figure suffers from an obvious circadian confound but the experiment in figure 2 does not. Indeed it is not clear what figure 1 added to the story, though some use for a subset of readers may come from it.

Authors' response: We thank the reviewer for his/her general appraisal of our findings and for the specific comments. We now provide more information on our reasoning to perform two independent experiments to untangle circadian from sleep-related effects. Accordingly, we have rewritten the last paragraph of our introduction (page 4) as follows:

"We investigated the role of sleep, and of distinct sleep stages, in the down-regulation of excitatory glutamatergic synapses in the rat hypothalamus. To corroborate and relate our results to previous findings (Vyazovskiy et al., 2008), we also assessed respective changes in the neocortex. We focused on postsynaptic AMPA receptors, specifically on the GluA1 and GluA2 subunits and the phosphorylation of GluA1 in synaptoneurosomes, as key substrates of synaptic renormalization and plasticity at excitatory synapses (Collingridge et al., 2004; Malenka and Bear, 2004; Diering and Huganir, 2018). These two subunits are also the most prominent AMPAR subunits expressed in rat hypothalamus and neocortex (Royo et al., 2022). We conducted two independent experiments to disentangle sleep-specific from potential circadian effects. In experiment 1, we compared a spontaneous wake group, in which tissue for synaptoneurosome analyses was obtained at 24.00 of the active phase, with a sleep group, in which tissue was obtained at 12.00 of the inactive phase, resulting in a between-group circadian shift of twelve hours. In experiment 2, we compared three groups, i.e., sleep, total sleep deprivation (TSD), and REM sleep deprivation (REM-D), which were all sacrificed at precisely the same time as the sleep group of experiment 1 (12.00 of the inactive phase), effectively eliminating any circadian influence. We found that independent of the circadian rhythm, sleep compared to wakefulness leads to a distinct decrease in the synaptic AMPA receptor subunit GluA1 in the hypothalamus, and that the reductions in hypothalamic GluA1 subunits due to sleep parallel those found in neocortex. Sleep-associated attenuations of GluA1-containing AMPARs phosphorylated at Ser845 or Ser831 appeared to be generally enhanced by circadian rhythmicity. Decreases in AMPA receptor subunit expression were comparable after undisturbed sleep and selective deprivation of REM sleep, indicating that SWS is the main driver of synaptic down-regulation in hypothalamic networks."

2. It is surprising that REM theta predicts GluA1 levels despite Figure 2 showing lack of import for REM. I don't think this was discussed and it should be.

Authors' response: We thank the reviewer for this comment, which connects to the second comment brought forward by reviewer #3. We would like to underline that the association between REM theta energy and GluA1 subunit levels appeared in the analyses of experiment 1 (Figure 3D, right panel: black dotted line), but was not confirmed by the respective analyses of experiment 2 (red line). We have rephrased the respective paragraph of our result section (page 12) as follows to further clarify this point:

"In order to confirm or rebut spindle density, time spent in SWS, and REM sleep theta energy as predictors of subunit levels in hypothalamic synaptoneurosomes, we analyzed data from the Sleep group of experiment 2, where animals likewise experienced uninterrupted sleep for six hours prior to AMPAR subunit assessment. For this purpose, we subjected these parameters to stepwise linear regression analyses. Again, we found increases in total sleep time as well as SWS and REM sleep duration in the second compared to the first three hours of the experimental 6-hour interval (**Figure 3 B/C** and **Supplementary Figure S4**). Regression analyses confirmed spindle density as a factor predicting GluA1 subunit levels in hypothalamic synaptoneurosomes ($b = 19.624$, $SEM = 7.12$, $t(5) = 2.75$, $p < 0.05$, for the optimal model fit): spindle density during the second 3-hour interval and GluA1 subunit levels were positively associated ($r = 0.9135$, $p =$

0.0015; **Figure 3D**). This correlation was not observed for cortical levels of GluA1 subunits (r = -0.0563, p = 0.8947, z = 2.6669, p = 0.0077 for the difference between correlations in hypothalamus and neocortex). In contrast to spindle density, the negative associations of SWS duration and REM sleep theta energy with GluA1-AMPAR subunit levels observed in experiment 1, however, were not confirmed (**Figure 3D**). Note also that not only is the statistical link between high-amplitude REM theta activity and the downregulation of hypothalamic GluA1-containing AMPARs restricted to experiment 1, but that the absence of effects on GluA1-AMPA receptor regulation of selective REM sleep deprivation (compared to regular sleep) in experiment 2 indicates that SWS alone is sufficient for sleep-dependent synaptic renormalization."

3. Overall the work is appears to be well done and well presented.

Authors' response: Thank you again for reviewing and evaluating our manuscript and for your uplifting general take on our findings and our submission.

Reviewer #2

This manuscript covers an interesting and timely topic - namely, the neurobiological underpinnings of sleep loss-driven changes that might underlie sleep homeostasis, e.g. changes to the hypothalamus, which regulates sleep and other homeostatic processes. Critically, the experiments of this study do not constitute the first assessment of biochemical changes due to sleep loss in the hypothalamus - transcriptomic studies of this have been carried out since the mid-2000s (and using more precise methodology, see Mackiewicz et al., 2007). More importantly, the present studies are using very old methods to characterize such changes - using bulk biochemistry of whole hypothalamus. More recent studies have provided better resolution about the complex effects of sleep loss on this VERY complex brain region - including single-cell transcriptomics and spatial transcriptomics. As such, there isn't any new epiphany in the present paper in terms of understanding neurobiological changes related to sleep or sleep loss. Beyond that, there are significant technical concerns that muddy the waters in terms of data interpretation, described below:

1. Experiment 1 uses circadian timepoints, so it is really impossible to make any conclusion about the relative importance of sleep vs. circadian timepoints. This is EXTREMELY significant as a caveat for studying the hypothalamus, in particular, due to the many hypothalamic structures engaged by the SCN circadian clock... which could easily drive any of the changes observed.

Authors' response: We thank the reviewer for his/her remarks and criticisms, which have clearly helped us to improve our manuscript. We have taken great care to clarify specific points and highlight key details of our analytical approach. We would like to emphasize that our strategy of conducting two independent experiments aimed precisely at disentangling circadian from sleep-specific effects. In experiment 1, we employed a spontaneous wake group as a control, which, in comparison with the sleep group, experienced a 12-hour shift in circadian phase. As the reviewer points out, the resulting differences between both groups may therefore, at least in part, reflect circadian effects. Therefore, we conducted experiment 2, in which we compared three groups (sleep, total sleep deprivation [TSD], and REM sleep deprivation [REM-D]) that were sacrificed at precisely the same time of day, effectively eliminating any circadian influence. This approach enabled us to differentiate circadian and sleep-dependent effects and moreover enhanced the reliability of correlational analyses. As outlined in our response to remark 1 from reviewer 1, we are now describing the rationale behind our study design in greater detail in the last paragraph of the introduction (page 4):

"We investigated the role of sleep, and of distinct sleep stages, in the down-regulation of excitatory glutamatergic synapses in the rat hypothalamus. To corroborate and relate our results to previous findings (Vyazovskiy et al., 2008), we also assessed respective changes in the neocortex. We focused on postsynaptic AMPA receptors, specifically on the GluA1 and GluA2 subunits and the phosphorylation of GluA1 in synaptoneurosomes, as key substrates of synaptic renormalization and plasticity at excitatory synapses (Collingridge et al., 2004; Malenka and Bear, 2004; Diering and Huganir, 2018). These two subunits are also the most prominent AMPAR subunits expressed in rat hypothalamus and neocortex (Royo et al., 2022). We

conducted two independent experiments to disentangle sleep-specific from potential circadian effects. In experiment 1, we compared a spontaneous wake group, in which tissue for synaptoneurosome analyses was obtained at 24.00 of the active phase, with a sleep group, in which tissue was obtained at 12.00 of the inactive phase, resulting in a between-group circadian shift of twelve hours. In experiment 2, we compared three groups, i.e., sleep, total sleep deprivation (TSD), and REM sleep deprivation (REM-D), which were all sacrificed at precisely the same time as the sleep group of experiment 1 (12.00 of the inactive phase), effectively eliminating any circadian influence. We found that independent of the circadian rhythm, sleep compared to wakefulness leads to a distinct decrease in the synaptic AMPA receptor subunit GluA1 in the hypothalamus, and that the reductions in hypothalamic GluA1 subunits due to sleep parallel those found in neocortex. Sleep-associated attenuations of GluA1-containing AMPARs phosphorylated at Ser845 or Ser831 appeared to be generally enhanced by circadian rhythmicity. Decreases in AMPA receptor subunit expression were comparable after undisturbed sleep and selective deprivation of REM sleep, indicating that SWS is the main driver of synaptic down-regulation in hypothalamic networks."

2. There is a large amount of variability of sleep amounts in both circadian time point groups in Experiment 1 (unsurprisingly). Critically, there is no attempt made to take advantage of this variability, for clarifying the data, as there could be a correlation between sleep time and the biochemical measurements.. this would actually make for a much better argument than the group differences shown.

Authors' response: We thank the reviewer for this helpful comment. He/she is right in pointing out that, as expected, sleep duration varied across individual animals in both the Sleep and the Wake groups of experiment 1 (Figure 1B). We made use of this variation in our correlational analyses. These analyses yielded significant correlations of SWS and REM duration in the second 3-hour interval with hypothalamic GluA1 downregulation in the Sleep group (**Supplementary Table 1**). Combining both samples in the correlational analyses yields a pronounced bimodal pattern, reflecting the different experimental groups, as exemplified in Response Figure R1, left panel, for SWS duration. In an exploratory analysis, we conducted a Spearman correlation across both groups of animals and found a significant negative correlation between SWS duration and GluA1 subunit levels ($r = -0.624$; $p < 0.01$), which, however, failed to reach significance in the Wake group alone ($p > 0.35$). Please note that these significances were not corrected for multiple testing, and we would be hesitant to report such findings in the manuscript unless they can be validated by subsequent analysis on independent data, as done for the other correlations presented in the manuscript.

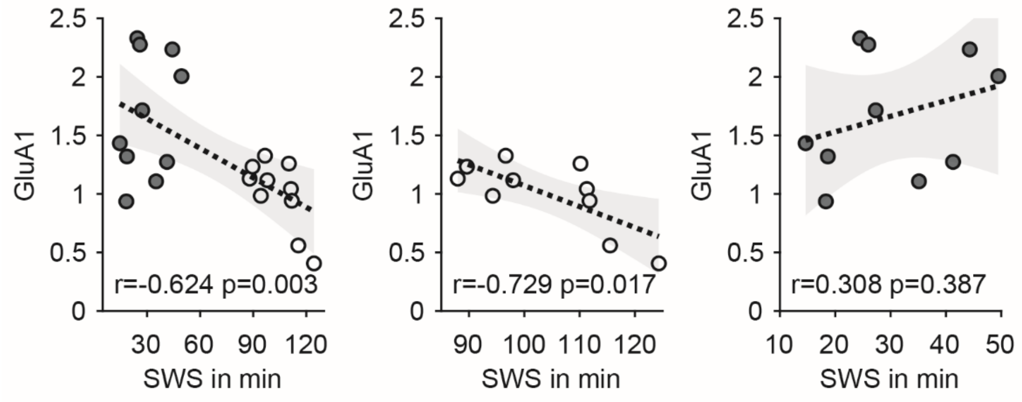

Response Figure R1: Correlations between GluA1 levels in the hypothalamus and time spent in SWS during the last 3 hours of sleep of experiment 1. Correlations are shown combined for animals of the Wake (grey circles) and Sleep groups (white circles) (left panel), of the Sleep group (middle panel), and of the Wake group (right panel).

As reported in the manuscript, we were unable to confirm the SWS-GluA1 correlation in the data of experiment 2. Given these dynamics, we opted for a stepwise regression approach to reliably investigate the relationship between electrophysiological markers of sleep and biochemical measurements, which provides

most robust compensation for multiple comparisons. Interestingly, spindle density emerged as the sole predictive factor for hypothalamic GluA1 levels.

3. In the schematic illustrations showing the region sampled for hypothalamic measures, it is striking that only a small ventral most portion of the hypothalamus seems to be highlighted. Rather, it looks like the SCN is being sampled! Is this an error in making the schematic?

Authors' response: We thank the reviewer for this helpful remark and would like to apologize for any confusion that the illustrations of neocortex and hypothalamus in Figures 1 and 2 might have caused – they were indeed only intended for a schematic illustration of both sites. We have now exchanged the illustration with a more anatomically correct illustration of the sampled tissue in Figures 1 and 2:

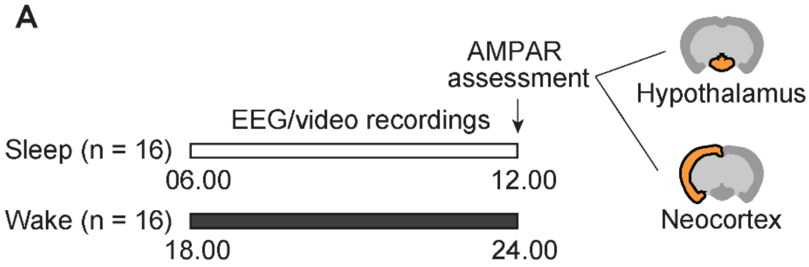

Figure 1. AMPAR levels in hypothalamus and neocortex after sleep and wakefulness. (**A**) Experimental design: AMPAR subunit levels were measured in synaptoneurosomes sampled from the entire hypothalamus and from the left cortical hemisphere of rats after experimental 6-hour periods taking place either during the animals' daytime rest period (starting at 6.00 h, lights on) or nighttime active period (starting at 18.00 h, lights off) and, accordingly, filled with spontaneous sleep (Sleep group, S; n = 16; white bars) or with wakefulness (Wake group, W; n = 16; black bars). During the 6-hour interval, animals were food-deprived while water was available ad libitum, and sleep was assessed by visually scoring behaviour (in 6 rats) and by EEG and EMG recordings (in 10 rats). […]

4. In the experiment where sleep is actually deprived (Experiment 2) many of the biochemical measures that were significantly different between groups (including, critically, the phosphorylations that are so important for synaptic potentiation) are NOT affected. This is true for both hippocampus AND neocortex! So.. those changes emphasized in Experiment 1 are effects of the circadian timepoints, rather than effects of sleep? If that is NOT the interpretation, what is it? It isn't clear what else could possibly make sense. So, the whole title and premise of the first part of the paper seem very misleading.

Authors' response: These important comments and questions are very much appreciated because they help us to clarify relevant aspects of our findings. While we acknowledge the significance of AMPAR phosphorylation in synaptic potentiation, we want to stress that changes in AMPAR density represent the primary measure we hypothesized to be influenced by sleep. This is based on the crucial distinction between AMPAR phosphorylation, which is a relatively rapid process, and the incorporation of AMPARs into the synapse, which reflects a more enduring remodeling of synaptic structure and connectivity. Consequently, AMPAR phosphorylation is subject to faster fluctuations, and studies on the role of AMPAR phosphorylation during homeostatic scaling suggest that dephosphorylated receptors are preferentially removed (Diering and Huganir, Neuron 2018).

We deliberately refer only to synaptic receptor levels in the title and throughout our manuscript to avoid the suggestion that sleep influences AMPAR phosphorylation levels. Indeed, accumulating evidence indicates that AMPAR phosphorylation may be influenced by circadian factors (Brüning et al., 2019; Liston et al., 2013). Additionally, the work by Vyazovskiy and colleagues (2008) demonstrated differences in AMPAR phosphorylation within the cortex for groups with varying circadian phases. To clarify possible circadian

contributions to the observed effects, we have extended the statistical comparison between experiment 1 and 2 for each of the proteins using a 2 (Experiment 1/2) × 2 (Hypothalamus/Neocortex) ANOVA approach. (Checking our original analyses, we detected an error in the analyses of hypothalamic GluA Ser 845 levels, which we have now corrected). Whereas we did not find any significant indicators of circadian influences for GluA1 and GluA2 levels ($p > 0.065$), we found significant main effects of the factor "*experiment (1/2)*" for Ser845 and Ser831, which supports the view that circadian effects contribute to the phosphorylation of GluA1 AMPARs in the cortex and hypothalamus. We are now providing a detailed account of these findings in the results and discussion sections as follows:

We have integrated these analyses as follows:

Results (page 9): "In experiment 1, the 6-hour experimental sleep and wake periods took place during the animals' rest and activity phases, respectively, and thus confounded effects of sleep and circadian rhythm, whereas in experiment 2, the experimental 6-hour periods before AMPAR assessment took place at the same circadian phase in all experimental groups, i.e., between 6.00 and 12.00 a.m. Accordingly, additional analyses comparing the effects of undisturbed sleep relative to wakefulness (i.e., with the values of the Wake and TSD groups in experiments 1 and, respectively, 2, set to 100%), which covered experiments 1 vs. 2 and both sites of interest (hypothalamus vs. neocortex), allowed the differentiation of potential circadian influences from effects of sleep. These ANOVA comparisons did not yield robust differences between experiments in the sleep effect on GluA1 and GluA2 levels (all $p > 0.065$), but revealed a main effect of *Experiment* (1 vs. 2) for the levels of GluA1 subunits phosphorylated at Ser845 and Ser831 ($F(1,16) = 14.804$, $p < 0.01$ and $F(1,16) = 9.157$, $p < 0.01$ respectively; $p > 0.106$ for respective *Experiment* × *Hypothalamus/Neocortex* interactions). Post-hoc analyses confirmed more pronounced decreases (compared to Wake and, respectively, TSD) in the Sleep groups of experiment 1 than experiment 2 for hypothalamic levels of GluA1 phosphorylated at Ser845 (60.930 ± 8.705% vs 103.846 ± 6.442%, $t(16) = -3.785$, $p < 0.01$) and for neocortical levels of GluA1 phosphorylated at Ser831 (67.42 ± 8.145% vs 104.432 ± 8.807%, $t(16) = -3.085$, $p < 0.01$). This pattern indicates a predominant role of sleep rather than circadian factors for the regulation of GluA1 and GluA2 levels, whereas circadian rhythmicity might add to the effect of sleep on GluA1 subunit phosphorylation."

Accordingly, we adapted the Discussion (page 17) as follows: "To dissociate effects of sleep from those of the circadian rhythm, we compared AMPAR subunit levels between experiment 1 (in which sleep and wakefulness occurred at opposing phases of the circadian cycle) and experiment 2 (in which the circadian phase was kept constant across the three groups). These analyses confirmed that the pronounced decreasing effect of sleep on GluA1-containing AMPARs emerges independently of any circadian influence. In some contrast, circadian rhythmicity significantly contributed to the decrease in the levels of phosphorylated GluA1-containig AMPARs. These findings are in line with evidence that the phosphorylation of GluA1 AMPARs is, to a certain extent, controlled by circadian clocks and may therefore occur at least in part independently of synaptic network plasticity associated with learning and memory processes during sleep and wakefulness (Bruning et al., 2019; Noya et al., 2019)."

5. Figure 3D - Two very major questions here. First, why is this the first attempt at a correlation? Second, why are all the animals' data not included in this correlation (it appears to be about half of the data points from animals with PSG analysis!!)?

Authors' response: This point connects to comment #2 from the same reviewer, and we thank the reviewer for the opportunity to further clarify our systematic approach to the correlational analyses of our findings. The results of the initial analysis of experiment 1 is summarized in **Supplementary Table S1,** which presents Pearson correlations with GluA1-containing AMPARs in hypothalamus and neocortex for relevant sleep parameters (such as SWS and REM duration, spindle density, SWA power, etc.). To investigate the relevance of these correlations, we performed stepwise regression analyses of the data collected in experiment 2. Figure 3D shows that these analyses confirmed the positive associations between spindle density and hypothalamic GluA levels (left panel), whereas the negative correlation between REM theta energy and hypothalamic GluA1 was not confirmed (right panel). Note that in experiment 2, we intentionally focused only on the those sleep parameters that had shown significant correlations in experiment 1 (SWS duration, spindle density, REM duration, and REM theta energy). In sum, while spindle density emerged as the sole predictor from the

stepwise regressions, the association of REM theta energy with GluA1 levels only reached significance in experiment 1. As outlined in our response to the second comment from the same reviewer, we refrained from correlating merged EEG data of Wake and Sleep groups due to the ensuing bimodal distribution. However, in light of the current comment, we have now reanalyzed all associations including data from both awake and sleeping animals by means of Spearman correlations. Notably, none of the correlations exhibited increased strength or significance.

We now highlight our approach in the legend of Figure 3: "…Data of experiment 2 were used to validate, via stepwise regression models, significant correlations identified in experiment 1."

Also, we provide greater detail in the Methods section (page 23): "… Linear correlation analyses between individual AMPAR subunit levels (expressed as percent values) and sleep parameters of interest relied on Pearson's product-moment correlation coefficients. Step-wise regression analysis of data from experiment 2 was employed to confirm or rebut significant correlations obtained in the analyses of experiment 1."

6. A general question - since sleep deprivation is known to alter the actin cytoskeleton, is using β-actin as a control for Westerns a very good idea? It seems like there might be a better choice, and that making the wrong choice could give a false impression.

Authors' response: This is an important methodological aspect, and we are thankful to the reviewer for providing us with the opportunity to address this issue. Sleep can indeed influence cytoskeletal proteins, as found, for example, in work conducted by Rodríguez-Vázquez et al. (2012) who utilized Western blotting on TAU and MAP2 proteins as markers of the neural cytoskeleton. Mackiewicz et al. (2007) quantified gene expression levels, observing that sleep alters the expression of genes involved in the nuclear transport of cytoskeleton complexes. However, to the best of our knowledge, there is no literature directly comparing changes in β-actin levels in synaptosomes between sleep and sleep deprivation or spontaneous wakefulness. Drawing on the dynamics observed in TAU and MAP2, and considering the concept of synapse remodeling during sleep potentially decreasing β-actin levels, we have conducted a direct comparison of β-actin levels collected in parallel with all proteins of interest (GluA1, GluA2, Ser845, and Ser831). In our synaptosome preparations, we did not observe any significant differences between the respective experimental groups in either of the experiments ($p > 0.59$).

We are now addressing this important point in the results section on pages 5-6:

"Control analyses of AMPAR subunit levels in supernatants did not indicate any detectable differences between the Sleep and Wake groups in hypothalamic or cortical samples (**Supplementary Figure S2**), confirming that the observed sleep-associated decreases in synaptoneurosomal GluA1 and GluA2 subunits are specific to the synapses. Importantly, to exclude any potential confounding effect of normalization to β-actin bands, we also directly compared β-actin levels collected from synaptoneurosomes and found that they did not differ between groups (hypothalamus: $t(78) = 0.531$, $p > 0.597$; neocortex: $t(78) = -1.318$, $p > 0.191$)."

On page 9, we now state: "Control analyses of supernatants and β-actin levels did not reveal any differences between the three groups, corroborating that the observed changes in AMPAR levels were specific to the synaptoneurosomes (**Supplementary Figure S3**) and, furthermore, not related to changes in β-actin levels (hypothalamus: $F(2,93) = 0.099$, $p > 0.905$) ; neocortex: $F(2,93) = 1.100$, $p > 0.336$)."

Reviewer #3

This is an interesting and impactful study showing that sleep leads to overall synaptic weakening also in the hypothalamus, which is an important novel finding. Liu and colleagues also provide novel evidence that NREM sleep alone is sufficient for synaptic renormalization to occur, both in cortex and hypothalamus. The results are solid and the paper well written, pending a few clarifications and qualifications for some statements that I believe are currently too strong, as indicated below.

Authors' response: We thank the reviewer for his/her general remarks on our manuscript; we are delighted that our findings are deemed to be of interest and to add novel insights to the field of sleep and synaptic homeostasis. We have clarified the aspects highlighted by the reviewer and have revised the manuscript with more balanced statements.

1. Line 81: the text and legend of figure 1a mentioned "filled with sleep or wake"; in reality, in both groups the 6 hours were not "filled" with just one behavioral state; please rephrase. In fact, figure 1B shows that several rats only slept 2.5-3 hours during the first half of the day, which is quite low. Based on figure 3B, most sleep happens in the last 3 hours, which raises the question whether these animals were well adapted to the light/dark cycle. Any explanation? Since it seems that there were no selection criteria before collecting the brains (i.e. even rats that slept less than 50% of the 6 hours were used), it would be useful to report (in a table?) the % of sleep and waking in the last 3 vs 6 hours before sacrifice, to see how they differ between the day and night group. Also (see below) the authors should report % of NREM and REM sleep separately.

Authors' response: We thank the reviewer for this comment. To ensure consistency between experiments 1 and 2, all animals underwent thorough habituation including exposure to the experimental recording box on three consecutive days (6 hours/d) prior to the experiment, guaranteeing comparable conditions across animals. Typically, automated sleep scoring assessments yield longer sleep durations compared to manual scoring, as they often allocate significant amounts of quiet wakefulness to sleep. To mitigate such potential confounds, we relied entirely on manual scorings.

As requested here and in point #10 below, in a new **Supplementary Figure S4,** we now show
1) an hour-by-hour analysis of SWS and REM sleep in both experiments and
2) a direct comparison of SWS and REM sleep durations during the final three hours between experiments 1 and 2 (in addition to Figure 3B, which depicts total sleep time (TST), SWS and REM durations in the Sleep groups during the first and second 3-hour intervals).

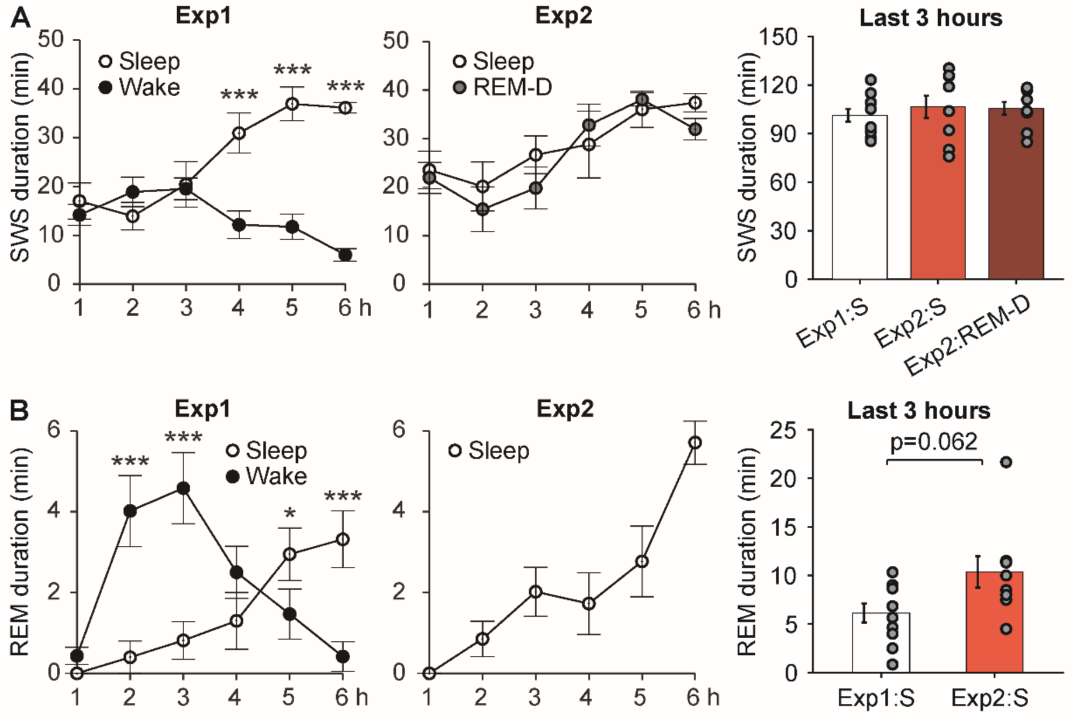

Supplementary Figure S4: Sleep architecture across the 6-hour recording intervals. Amount of (**A**) SWS and (**B**) REM sleep in minutes during each hour of the 6-hour recording session in the Sleep and Wake groups of experiment 1 (left panels) and in the Sleep and, as applicable, REM sleep-deprivation (REM-D) groups of experiment 2 (midline panels); SWS and REM sleep duration in minutes during the final 3 hours in the Sleep

(Exp1:S and Exp2:S respectively) and, as applicable, REM-D groups in experiments 1 and 2 (right panels); *** $p < 0.001$, unpaired t-tests. Note that overall, the sleep ratios are very much comparable between the respective groups of experiments 1 and 2.

2. The authors state that the results of " experiment 2 clearly rules out any causal contribution of REM sleep" and "SWS is the main driver" for sleep-dependent synaptic downselection. I find these results convincing. But, I am not prepared to dismiss REM sleep, especially after seeing figure 3A; if this is representative, there is little REM sleep in these rats, perhaps not surprisingly since several of these animals slept mainly in the last 3 hours (?), and the experiment is in the first half of the day; in short, I think from the current results we can conclude that NREM sleep alone is sufficient for sleep-dependent down-selection, but not that REM sleep has no role in it.

Authors' response: This comment on the relevance of REM sleep in comparison to NREM sleep is very much appreciated; it also connects to the second remark by reviewer #1 (see above). We have replaced the example hypnogram shown in Figure 3A with a more representative example of the typical distribution of SWS and REM sleep in the Sleep group of experiment 1, and moreover added an example hypnogram from the Wake group of experiment 1.

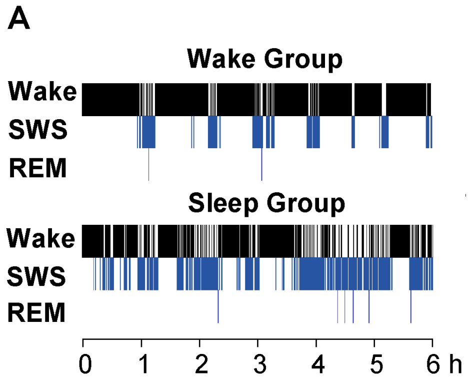

Figure 3. Spindle density predicts GluA1 AMPAR subunit levels in the hypothalamus. (A) Hypnogram for the 6-hour period before assessment of AMPAR subunits obtained in an individual rat of the Wake (top) and Sleep (bottom) group of experiment 1 (SWS, slow wave sleep; REM, rapid eye movement sleep). …"

As suggested by the reviewer, we have also rephrased the corresponding paragraph of our results section (page 12): "In contrast to spindle density, the negative associations of SWS duration and REM sleep theta energy with GluA1-AMPAR subunit levels observed in experiment 1, however, were not confirmed (Figure 3D). Note also that not only is the statistical link between high-amplitude REM theta activity and the downregulation of hypothalamic GluA1-containing AMPARs restricted to experiment 1, but that the absence of effects on GluA1-AMPA receptor regulation of selective REM sleep deprivation (compared to regular sleep) in experiment 2 indicates that SWS alone is sufficient for sleep-dependent synaptic renormalization."

3. The authors interpret the positive correlation between spindle activity and AMPAR expression in the hypothalamus as supporting evidence that spindles promote memory consolidation, but it is difficult to understand why this correlation is not found in the cortex. Spindles are thalamocortical events: why would they contribute to synaptic potentiation in the hypothalamus but not in the cortex? Indeed most spindles, like slow waves, are local, but across several hours, spindles should presumably affect most of the cortex…. How (via which anatomical pathways) would spindles reach the hypothalamus?

Authors' response: We thank the reviewer for this comment. We now discuss this finding in greater detail in the discussion on page 17: "… Noteworthy, in the present experiments a robust association with spindles was observed for GluA1-containing AMPAR levels in hypothalamic, but not in neocortical synaptoneurosomes. One reason for this might be that in the neocortex, up-regulating effects of spindles on GluA1 AMPARs are

primarily conveyed by locally acting spindles (Ujma et al., 2021; Weber et al., 2021), whereas we assessed the link between EEG spindles recorded at only a single electrode site and global AMPAR levels in an entire neocortical hemisphere. Moreover, in the neocortex the number of cells that are activated during a spindle is relatively low and fluctuates significantly across consecutive SWS episodes (Niethard et al., 2021). It is therefore plausible to assume that spindles in neocortical networks only affect a relatively small fraction of AMPARs. Our findings suggest a different scenario for hypothalamic networks, in which spindles seem to exert a more widespread effect, regulating AMPAR levels throughout the hypothalamus. While the presence of spindles in hypothalamic networks awaits confirmation, the pattern found here might well be in line with a role of hypothalamic circuitries in gating long-term memory formation (Kosse and Burdakov, 2019; Burdakov and Peleg-Raibstein, 2020). …"

4. Abstract, line 3: It is not correct to say that SHY "focuses on AMPA signaling"; SHY has been tested using many markers of synaptic strength, only one of which is the expression of AMPA receptors expression. Other markers include the size of the postsynaptic area measured with serial electron microscopy in axospinous (excitatory) synapses, and excitatory minis. In flies, SHY was tested using presynaptic (mainly cholinergic) markers, because the majority of synapses in flies are cholinergic.

Authors' response: We are happy to give a more balanced and refined account of SHY in the abstract.

"According to the Synaptic Homeostasis Hypothesis (SHY), sleep serves to re-normalize synaptic connections that have been potentiated during the prior wake phase due to ongoing encoding of information. SHY focuses on glutamatergic synaptic strength and has been supported by numerous studies examining synaptic structure and function in neocortical and hippocampal networks. However, it is unknown whether synaptic down-regulation during sleep occurs in the hypothalamus, i.e., a pivotal center of homeostatic regulation of bodily functions including sleep itself. We show that sleep, in parallel with the synaptic down-regulation in neocortical networks, down-regulates the levels of α-amino-3-hydroxy-5-methyl-4-isoxazolepropionic acid receptors (AMPARs) in the hypothalamus of rats.…"

5. Abstract, line 2 and 12 (and in many places in the main text, e.g. line 26 of the introduction): "global" can be misleading because it may be interpreted as saying that according to SHY, all synapses are weakened by sleep. According to SHY, the sleep-dependent weakening of synapses is not global in the sense that it does not affect every synapse; in fact, the process is selective (hence the term sleep-dependent down-selection), but broad (general), i.e. affecting most synapses and resulting in a net decrease in synaptic strength relative to waking; the fact that the process does not affect every single synapse was shown with serial EM (de Vivo 2017) and with single synapse resolution in Miyamoto et al., 2021.

Authors' response: We thank the reviewer for bringing up this relevant point, which we are gladly addressing by removing the wording "global" or "globally" in the respective sections of our manuscript.

Abstract: "According to the Synaptic Homeostasis Hypothesis (SHY), sleep serves to ~~globally~~ re-normalize synaptic connections that have been potentiated during the prior wake phase due to ongoing encoding of information. SHY focuses on glutamatergic synaptic strength and has been supported by numerous studies examining synaptic structure and function in neocortical and hippocampal networks. However, it is unknown whether synaptic down-regulation during sleep occurs in the hypothalamus, i.e., a pivotal center of homeostatic regulation of bodily functions including sleep itself. We show that sleep, in parallel with the synaptic down-regulation in neocortical networks, down-regulates the levels of α-amino-3-hydroxy-5-methyl-4-isoxazolepropionic acid receptors (AMPARs) in the hypothalamus of rats. Most robust decreases after sleep were observed at both sites for AMPARs containing the GluA1 subunit. Comparing the effects of selective REM sleep and total sleep deprivation, we moreover provide experimental evidence that slow wave sleep (SWS) is the driving force of the ~~global~~ down-regulation of AMPARs in hypothalamus and neocortex, with no additional contributions of REM sleep or the circadian rhythm. SWS-dependent synaptic down-regulation was not linked to EEG slow wave activity. However, spindle density during SWS predicted relatively increased GluA1 subunit levels in hypothalamic synapses, which is consistent with the role of spindles in the

consolidation of memory. Our findings identify SWS as the main driver of the renormalization of synaptic strength during sleep, and suggest that SWS-dependent synaptic renormalization is also implicated in homeostatic control processes in the hypothalamus.”

Introduction (page 3): “Experience during the wake phase is encoded into the brain’s neuronal networks by strengthening the synaptic connections in specific neuron ensembles. The encoding of information during wakefulness thus leads to a widespread strengthening of synaptic networks which, in the absence of counterregulatory processes, would ultimately yield a state of saturation. The synaptic homeostasis theory (SHY) proposes that sleep following the wake phase is the essential process that broadly renormalizes ~~global~~ synaptic strength (Tononi and Cirelli, 2014, 2020). Specifically, …“

Discussion (page 14): “… Overall, our results from neocortical synaptoneurosomes, in accordance with the earlier findings by Vyazovskiy et al. (2008) and in conjunction with studies employing various other structural and functional measures of network synaptic connectivity – like the size of axon-spine interfaces and spine heads determined by electron microscopy (de Vivo et al., 2017) and the amplitude of somatosensory and motor evoked potentials in humans (Huber et al., 2013) – corroborate the view proposed by SHY that, in the neocortex, network synaptic potentiation increases over periods of wakefulness, whereas sleep promotes a ~~global~~ depression and renormalization of synaptic strength..

6. Line 28-29: The sentence should be rephrased. SHY assumes that the net effect of waking is the strengthening of many excitatory synapses. Hundreds of studies by others have shown that this strengthening is reflected in increased expression of AMPARs. So "SHY assumes that wake encoding of information manifests itself mainly in the potentiation of excitatory synapses, which is known to result in increased numbers of ….."

Authors' response: We thank the reviewer for this comment and have changed this sentence accordingly (page 3): “…Specifically, SHY assumes that wake encoding of information manifests itself mainly in the potentiation of excitatory synapses, which is known to result in increased numbers of α-amino-3-hydroxy-5-methyl-4-isoxazole propionic acid (AMPA) type glutamate receptors, while they are down-regulated during subsequent sleep. This down-selection of potentiated synapses is assumed to be driven by the <1 Hz slow oscillation (SO) that hallmarks the stage of slow wave sleep (SWS).”

7. Line 62, 67, 74: "scaling" should be avoided, unless there is direct evidence for actual scaling.

Authors' response: We thank the reviewer for this remark. We have replaced the term scaling with more appropriate terminology throughout the manuscript. On page 4 for example, it now reads:

“We investigated the role of sleep, and of distinct sleep stages, in the down-regulation of excitatory glutamatergic synapses in the rat hypothalamus. To corroborate and relate our results to previous findings (Vyazovskiy et al., 2008), we also assessed respective changes in the neocortex. We focused on postsynaptic AMPA receptors, specifically on the GluA1 and GluA2 subunits and the phosphorylation of GluA1 in synaptoneurosomes, as key substrates of synaptic renormalization and plasticity at excitatory synapses (Collingridge et al., 2004; Malenka and Bear, 2004; Diering and Huganir, 2018). These two subunits are also the most prominent AMPAR subunits expressed in rat hypothalamus and neocortex (Royo et al., 2022). We conducted two independent experiments to disentangle sleep-specific from potential circadian effects. In experiment 1, we compared a spontaneous wake group, in which tissue for synaptoneurosome analyses was obtained at 24.00 of the active phase, with a sleep group, in which tissue was obtained at 12.00 of the inactive phase, resulting in a between-group circadian shift of twelve hours. In experiment 2, we compared three groups, i.e., sleep, total sleep deprivation (TSD), and REM sleep deprivation (REM-D), which were all sacrificed at precisely the same time as the sleep group of experiment 1 (12.00 of the inactive phase), effectively eliminating any circadian influence. We found that independent of the circadian rhythm, sleep compared to wakefulness leads to a distinct decrease in the synaptic AMPA receptor subunit GluA1 in the hypothalamus, and that the reductions in hypothalamic GluA1 subunits due to sleep parallel those found in neocortex. Sleep-associated attenuations of GluA1-containing AMPARs phosphorylated at Ser845 or Ser831

appeared to be generally enhanced by circadian rhythmicity. Decreases in AMPA receptor subunit expression were comparable after undisturbed sleep and selective deprivation of REM sleep, indicating that SWS is the main driver of synaptic down-regulation in hypothalamic networks."

8. Line 82-84: the authors should refer here to suppl figure 3, where they provide evidence that their synaptoneurosome preparation is enriched in synaptic proteins.

Authors' response: We have now introduced a reference to **Supplementary Figure S1** (previously Supplementary Figure S3) as follows on page 5: "Synaptoneurosomes are enriched in synaptic proteins and, thus, optimal for detecting activity-dependent changes in glutamate receptor levels (see also Methods/Preparation of synaptoneurosomes and **Supplementary Figure S1**)."

9. Line 96: please rephrase: the cortical changes do not really "correspond" to those in the hypothalamus (GluA2 and Ser831 change in cortex only).

Authors' response: We thank the reviewer for this remark. The corresponding sentence now reads (page 5): "In the neocortex, we detected differences between the Sleep and Wake groups in the levels of GluA1- and GluA2-containing AMPARs in synaptoneurosomes and in GluA1 phosphorylated at Ser845 and Ser831. Compared with the Wake group, the Sleep group…"

10. Figure 2B: same issue as in figure 1B. Several S rats are sleeping around or less than 50% of the first 6 hours of the day, so it would be useful to know the distribution and % of sleep and waking in the last 3 hours before sacrifice, to see how they differ across groups.

Authors' response: We thank the reviewer for bringing up this point. As outlined in our response to comment #1 by this reviewer, we have added a new **Supplementary Figure S4** illustrating the respective durations for each of the six consecutive hours for the sleep, wake, and REM deprivation groups for experiment 1 and 2.

11. Figure 2C: the lack of hypothalamic changes in Ser845 is a bit surprising and not discussed.

Authors' response: This important comment relates to point #4 from reviewer #2, and we thank both reviewers for addressing the issue of phosphorylation, also in light of potential circadian contributions. As outlined above, we have thoroughly assessed differences between the results of both experiments that may imply a role of circadian factors and present them in the Results on page 9; they are discussed pages 17-18.

Results (page 9): "In experiment 1, the 6-hour experimental sleep and wake periods took place during the animals' rest and activity phase, respectively, and thus confounded effects of sleep and circadian rhythm, whereas in experiment 2, the experimental 6-hour periods before AMPAR assessment took place at the same circadian phase in all experimental groups, i.e., between 6.00 and 12.00 a.m. Accordingly, additional analyses comparing the effects of undisturbed sleep relative to wakefulness (i.e., with the values of the Wake and TSD groups in experiments 1 and, respectively, 2, set to 100%), which covered experiments 1 vs. 2 and both sites of interest (hypothalamus vs. neocortex), allowed the differentiation of potential circadian influences from effects of sleep. These ANOVA comparisons did not yield robust differences between experiments in the sleep effect on GluA1 and GluA2 levels (all $p > 0.065$), but revealed a main effect of *Experiment* (1 vs. 2) for the levels of GluA1 subunits phosphorylated at Ser845 and Ser831 ($F(1,16) = 14.804$, $p < 0.01$ and $F(1,16) = 9.157$, $p < 0.01$ respectively; $p > 0.106$ for respective *Experiment* × *Hypothalamus/Neocortex* interactions). Post-hoc analyses confirmed more pronounced decreases (compared to Wake and, respectively, TSD) in the Sleep groups of experiment 1 than experiment 2 for hypothalamic levels of GluA1 phosphorylated at Ser845 (60.930 ± 8.705% vs 103.846 ± 6.442%, $t(16) = -3.785$, $p < 0.01$) and for neocortical levels of GluA1 phosphorylated at Ser831 (67.42 ± 8.145% vs 104.432 ± 8.807%, $t(16) = -3.085$, $p < 0.01$). This pattern indicates a predominant role of sleep rather than circadian factors for the regulation of GluA1 and GluA2 levels, whereas circadian rhythmicity might add to the effect of sleep on GluA1 subunit phosphorylation."

Discussion (pages 17-18): “To dissociate effects of sleep from those of the circadian rhythm, we compared AMPAR subunit levels between experiment 1 (in which sleep and wakefulness occurred at opposing phases of the circadian cycle) and experiment 2 (in which the circadian phase was kept constant across the three groups). These analyses confirmed that the pronounced decreasing effect of sleep on GluA1-containing AMPARs emerges independently of any circadian influence. In some contrast, circadian rhythmicity significantly contributed to the decrease in the levels of phosphorylated GluA1-containig AMPARs. These findings are in line with evidence that the phosphorylation of GluA1 AMPARs is, to a certain extent, controlled by circadian clocks and may therefore occur at least in part independently of synaptic network plasticity associated with learning and memory processes during sleep and wakefulness (Bruning et al., 2019; Noya et al., 2019).”

12. Line 110: more correctly, the authors show that AMPAR expression is higher after total SD than after sleep, not that SD increased it.

Authors' response: We are thankful for this comment and have rephrased the heading of this paragraph as follows (page 7): “AMPAR expression is higher after total sleep deprivation, but unchanged after REM sleep deprivation compared to sleep.”

13. Line 333: typo in AMPAR levels

Authors' response: Thank you – we have corrected this typo.

References:

Brüning, F., Noya, S. B., Bange, T., Koutsouli, S., Rudolph, J. D., Tyagarajan, S. K., ... & Robles, M. S. (2019) Sleep-wake cycles drive daily dynamics of synaptic phosphorylation. Science, 366(6462), eaav3617.

Burdakov D, Peleg-Raibstein D (2020) The hypothalamus as a primary coordinator of memory updating. Physiol Behav 223:112988.

Collingridge GL, Isaac JT, Wang YT (2004) Receptor trafficking and synaptic plasticity. Nature Reviews Neuroscience 5:952-962.

de Vivo L, Bellesi M, Marshall W, Bushong EA, Ellisman MH, Tononi G, Cirelli C (2017) Ultrastructural evidence for synaptic scaling across the wake/sleep cycle. Science 355:507-510.

Diedenhofen, B., & Musch, J. (2015) cocor: A comprehensive solution for the statistical comparison of correlations. PloS one, 10(4), e0121945.

Diering GH, Huganir RL (2018) The AMPA Receptor Code of Synaptic Plasticity. Neuron 100:314-329.

Huber R, Maki H, Rosanova M, Casarotto S, Canali P, Casali AG, Tononi G, Massimini M (2013) Human cortical excitability increases with time awake. Cereb Cortex 23:332-338.

Kosse C, Burdakov D (2019) Natural hypothalamic circuit dynamics underlying object memorization. Nat Commun 10:2505.

Liston, C., Cichon, J. M., Jeanneteau, F., Jia, Z., Chao, M. V., & Gan, W. B. (2013) Circadian glucocorticoid oscillations promote learning-dependent synapse formation and maintenance. Nature neuroscience, 16(6), 698-705.

Mackiewicz, M., Shockley, K. R., Romer, M. A., Galante, R. J., Zimmerman, J. E., Naidoo, N., ... & Pack, A. I. (2007) Macromolecule biosynthesis: a key function of sleep. Physiological genomics, 31(3), 441-457.

Malenka RC, Bear MF (2004) LTP and LTD: an embarrassment of riches. Neuron 44:5-21.

Miyamoto D, Marshall W, Tononi G, Cirelli C (2021) Net decrease in spine-surface GluA1-containing AMPA receptors after post-learning sleep in the adult mouse cortex. Nat Commun 12:2881.

Niethard N, Brodt S, Born J (2021) Cell-Type-Specific Dynamics of Calcium Activity in Cortical Circuits over the Course of Slow-Wave Sleep and Rapid Eye Movement Sleep. J Neurosci 41:4212-4222.

Noya SB, Colameo D, Brüning F, Spinnler A, Mircsof D, Opitz L, Mann M, Tyagarajan SK, Robles MS, Brown SA (2019) The forebrain synaptic transcriptome is organized by clocks but its proteome is driven by sleep. Science 366.

Rodríguez-Vázquez, J., Camacho-Arroyo, I., & Velázquez-Moctezuma, J. (2012) Differential impact of REM sleep deprivation on cytoskeletal proteins of brain regions involved in sleep regulation. Neuropsychobiology, 65(3), 161-167.

Royo M, Escolano BA, Madrigal MP, Jurado S (2022) AMPA Receptor Function in Hypothalamic Synapses. Front Synaptic Neurosci 14:833449.

Tononi G, Cirelli C (2014) Sleep and the price of plasticity: from synaptic and cellular homeostasis to memory consolidation and integration. Neuron 81:12-34.

Tononi G, Cirelli C (2020) Sleep and synaptic down-selection. Eur J Neurosci 51:413-421.

Ujma PP, Hajnal B, Bodizs R, Gombos F, Eross L, Wittner L, Halgren E, Cash SS, Ulbert I, Fabo D (2021) The laminar profile of sleep spindles in humans. Neuroimage 226:117587.

Vyazovskiy VV, Cirelli C, Pfister-Genskow M, Faraguna U, Tononi G (2008) Molecular and electrophysiological evidence for net synaptic potentiation in wake and depression in sleep. Nat Neurosci 11:200-208.

Weber FD, Supp GG, Klinzing JG, Molle M, Engel AK, Born J (2021) Coupling of gamma band activity to sleep spindle oscillations - a combined EEG/MEG study. Neuroimage 224:117452.
